# UroVysion^™^ fluorescence *in situ* hybridization (FISH) possibly has a high positive rate in carcinoma of non-urothelial lineages

**DOI:** 10.3389/fmolb.2023.1250442

**Published:** 2023-10-05

**Authors:** Chunjin Ke, Xuguang Liu, Jie Wan, Zhiquan Hu, Chunguang Yang

**Affiliations:** ^1^ Department of Urology, Tongji Hospital of Tongji Medical College, Huazhong University of Science and Technology (HUST), Wuhan, China; ^2^ Department of Pathology, Tongji Hospital of Tongji Medical College, Huazhong University of Science and Technology (HUST), Wuhan, China

**Keywords:** fluorescence *in situ* hybridization, chromosome, CNUL, squamous cell carcinoma, adenocarcinoma

## Abstract

**Background:** Positive UroVysion^™^ fluorescence *in situ* hybridization (FISH) is generally considered as urothelial carcinoma (UC). We clarify if UroVysion^™^ FISH can be positive in carcinoma of non-urothelial lineages (CNUL), and verify the consistency of urine FISH and histological FISH in CNUL.

**Methods:** All CNUL subjects detected by urine FISH assay due to haematuria from Tongji Hospital were screened. Meanwhile, 2 glandular cystitis and 2 urothelial carcinoma were served as negative or positive control. Paraffin-embedded tissue sections of all subjects were sent to the pathology department for histological FISH detection.

**Results:** A total of 27 patients were included in this study, including 9 with adenocarcinomas, 11 with squamous cell carcinomas, and 7 with other tumour types. The overall positive rate in urine FISH was 64.00% (16/25) in patients with CNUL, 77.78% (7/9) in those with adenocarcinoma and 54.55% (6/11) in those with squamous carcinoma. There was a significant difference in the GLP p16 gene deletion rate between UC and CNUL (100% vs. 8.00%, *p* = 0.017). Histological FISH results showed that the histological results of 19 patients were consistent with their urine FISH results, and only one patient with stage Ⅲa urachal carcinoma had inconsistent histological FISH results (positive) and urine FISH (negative) results.

**Conclusion:** We demonstrated for the first time the application value of FISH in CNUL on urine samples. Positive urine FISH tests indicate not only UC, but also CNUL. UroVysion^™^ FISH possibly has a high positive rate in CNUL. CNUL and UC have different genetic changes shown by FISH.

## 1 Introduction

Fluorescence *in situ* hybridization (FISH) detects chromosomal or genetic abnormalities in cell and tissue samples by detecting fluorescence signals through fluorescence microscopy after hybridization between the probe and the DNA of the sample through the complementarity of DNA base pairs (Wiegant et al., 1991; Sokolova et al., 2000; Levsky and Singer, 2003; National Library of Medicine, 2020). The U.S. Food and Drug Administration approved UroVysion^™^ FISH probes (chromosomes 3, 7, and 17 combined with the 9p21 probe) in 2001 and 2005, respectively, for urine detection in patients with suspected bladder cancer and postoperative recurrence monitoring in patients with bladder cancer (Halling et al., 2000). Worldwide, the incidence of bladder cancer ranks 9th among all malignant tumours in the body and 7th among male patients. Regarding histopathological classification, more than 90% of cases are bladder urothelial carcinoma (Jemal et al., 2010; Siegel et al., 2017). In recent years, the incidence of bladder cancer in China has been increasing year by year, with an average growth rate of 68.29% in the past 15 years, due to changes in diet, increased work pressure, harsh environment and other factors (Chen, 2015; Chen et al., 2016). Therefore, FISH positive is usually considered to be urothelial carcinoma (UC).

A review of the national and international literature shows that there are very few studies on the application of FISH in carcinoma of non-urothelial lineages (CNUL). (Reid-Nicholson et al., 2009) performed histological FISH detection on paraffin sections from CNUL patients and found that FISH positivity was common in primary and secondary adenocarcinomas but rare in squamous cell carcinomas. (Kipp et al., 2008) also performed histological FISH on paraffin sections and found that chromosomal abnormalities detected in UC were common in rare histological types of bladder cancer. (Yang et al., 2016) found that preoperative urine FISH were positive in patients with bladder paraganglioma, which showed polyploidy of chromosomes 3 and 17. Urine FISH was performed again after the operation and the result turned negative. In our clinical practice, we successively found that urine FISH showed positive manifestations in urachal carcinoma (Case 8-Case 14), (Hu et al., 2020), renal secondary non-Hodgkin lymphoma (Case 7) and renal squamous cell carcinoma (Case 17), (Hu et al., 2021), so we did a comprehensive review of all the cases in our center since the FISH technique was introduced.

In summary, it is clear that the positive presentation of urine FISH in CNUL is not coincidental, however, none of the existing studies have been cross-validated by histological FISH with urine FISH, thus causing a lack of studies to demonstrate the relationship between the two specimen types. This study focuses on elucidating that FISH can also show positive in urine or tissue specimens of CNUL, thus suggesting that FISH-positive patients do not always have UC. The second is to confirm the consistency of histological and urine FISH analysis results to fill the gap of previous studies.

## 2 Methods

### 2.1 Research objects

With the approval of the Medical Ethics Committee of Tongji Hospital affiliated with Tongji Medical College, Huazhong University of Science and Technology (Approval No. TJ-IRB20210521), we applied to the Department of Pathology to query the information of patients with CNUL admitted to the Department of Urology in the past 10 years, including all squamous carcinomas, adenocarcinomas and other rare types of tumours. Then, we retrospectively searched the FISH database at our Institute of Urology for relevant FISH testing information for these patients. A total of 25 patients who met the requirements were screened, and 2 patients with UC were selected as the control group. Inclusion criteria were as follows: ① can obtain specific clinical data through the electronic medical record system; ② have histological specimens in our hospital and have been pathologically diagnosed as CNUL; ③ have no urinary calculi, chemotherapy, radiotherapy, etc.

### 2.2 Research methods

#### 2.2.1 Detection method of urine FISH

The specific results of urine FISH in 27 patients were obtained directly from the Institute of Urology of Tongji hospital. Approximately 200 ml of urine was collected in the morning. The volume of urine specimen should not be too small, otherwise it will affect the number of cells in the specimen and cannot meet the basic requirements of FISH detection technology. Urine specimens should be kept free of contaminants such as prostate fluid, semen, leukorrhea, menstrual blood, etc. After the specimen is collected, it is necessary to send it for examination as soon as possible to prevent the dissolution of cells in the specimen, resulting in changes in the composition of the specimen.

#### 2.2.2 Detection method of histological FISH

Haematoxylin and eosin-stained slides (and immunohistochemistry slides, if applicable) of the paraffin-embedded tissue of the relevant patient were first requested from the Department of Pathology, and then representative paraffin blocks containing ≥ 80% tumour cells were selected for histological FISH. The target areas for hybridization were highlighted on each representative slide. All metastatic tumours were confirmed by immunohistochemistry and/or review of the primary tumour. The FISH DNA probe kit (Bladder cancer cell chromosome and gene abnormality detection box: China Food and Drug Administration No. 3400251, 2009; Order number F01008-02) was purchased from Beijing Jinpujia Medical Technology Co. Ltd. The FISH DNA probe is labelled with tetramethylrhodamine and fluorescein isothiocyanate and consists of two combinations: CSP3 (green)/CSP7 (red) and GLP p16 (red)/CSP17 (green). For experimental procedures and result interpretation standards (Supplementary Material), refer to the published articles by our team (Hu et al., 2020) and the official website of the kit supplier company (National Library of Medicine, 2020).

### 2.3 Statistical analysis

Data analysis was performed using IBM SPSS Statistics^®^ version 23 (IBM Corp, Armonk, NY, USA) (Liang et al., 2019). Continuous variables are expressed as the median ± interquartile spacing, and count variables are described as frequencies, ratios and percentages. Categorical variables were compared using the chi-square test and Fisher’s exact test when data were limited. Differences with *p* < 0.05 were considered statistically significant.

## 3 Results

### 3.1 Urine FISH positive detection rate of CNUL

A total of 27 patients were included in this study: 9 with adenocarcinoma, 11 with squamous carcinoma, and 7 with other types [2 with glandular cystitis, 2 with UC (control group), 1 with small cell carcinoma of the bladder, 1 with renal secondary non-Hodgkin’s lymphoma, and 1 with bladder paraganglioma]. According to the pathological results of postoperative specimens and related clinical data, adenocarcinoma was further divided into 7 cases of urachal adenocarcinoma and 2 cases of prostate acinar carcinoma invading the bladder. Squamous cell carcinoma was divided into primary and secondary squamous cell carcinoma. The primary squamous cell carcinoma was divided into 6 cases of renal pelvis squamous cell carcinoma and 3 cases of bladder squamous cell carcinoma. Two secondary renal squamous cell carcinoma cases were derived from oesophageal squamous cell carcinoma and lung squamous cell carcinoma.

The above 27 patients were all subjected to urine FISH assay due to haematuria or suspected UC and other factors, and the collected urine samples all met the testing requirements. Of the 27 patients, 22 were males, and 5 were females, with a median age of 54 (50–65) years. The overall positive rate in urine FISH was 64.00% (16/25) in patients with CNUL. The positive rate of adenocarcinoma was 77.78% (7/9), including 5 cases of urachal carcinoma (71.43%, 5/7) and 2 cases of prostate acinar carcinoma invading the bladder (100%, 2/2). In squamous cell carcinoma, the positive rate was 54.55% (6/11), including 3 cases of primary pure bladder squamous cell carcinoma (100%, 3/3), 1 case of primary renal pelvis squamous cell carcinoma (16.67%, 1/6), and 2 cases of secondary renal squamous cell carcinoma (derived from oesophageal squamous cell carcinoma and lung squamous cell carcinoma).

For advanced or certain rare tumors, such as metastatic tumors (2 cases of metastatic renal squamous carcinoma, 1 case of metastatic non-Hodgkin’s lymphoma, and 1 case of urachal carcinoma with distant visceral metastasis), prostate cancer invading the bladder, and small cell carcinoma of the bladder, urine FISH is prone to be positive. Therefore, the more malignant and advanced the tumor is, the more likely it is to result in positive urine FISH (Table 1).

**TABLE 1:** Basic clinical data of 27 patients.

No	Age (years)	Sex	Diagnosis	Urine FISH (+/−)	Abnormal cell ratio (%)				Histological FISH (+/−)	Genetic material changes (+/−)			
					CSP3	CSP7	GLP p16	CSP17		CSP3	CSP7	GLP p16	CSP17
1	69	male	Urothelial carcinoma	+	47	47	45	51	+	+	+	+	+
2	64	female	Urothelial carcinoma	+			19	15	+	-	-	+	+
3	66	male	Small cell carcinoma of the bladder	+	83	86		85	+	+	+	-	+
4	34	male	Bladder paraganglioma	+	65			75					
5	50	female	Cystitis glandularis	-					-	-	-	-	-
6	53	male	Cystitis glandularis	-					-	-	-	-	-
7	50	male	Renal secondary non-Hodgkin lymphoma	+	63	63		58					
8	25	male	Urachal adenocarcinoma	+	39	47			+	+	+	-	-
9	49	female	Urachal adenocarcinoma	-					-	-	-	-	-
10	50	female	Urachal adenocarcinoma	-					+	-	+	-	+
11	68	male	Urachal adenocarcinoma	+	14	12							
12	46	male	Urachal adenocarcinoma	+	27	30	31	32					
13	54	male	Urachal adenocarcinoma with distant visceral metastasis	+	31	29							
14	30	male	Urachal adenocarcinoma	+	15	17		18					
15	69	male	Prostate cancer invades the bladder	+	33	35		31	+	+	+	-	+
16	72	male	Prostate cancer invades the bladder	+	67	69		67	+	+	+	-	+
17	56	male	Renal squamous cell carcinoma (oesophageal squamous cell carcinoma metastasis)	+	23	23		21	+	+	+	-	+
18	65	male	Renal squamous cell carcinoma	-					-	-	-	-	-
19	58	male	Renal squamous cell carcinoma	-					-	-	-	-	-
20	63	male	Renal squamous cell carcinoma (lung squamous cell carcinoma metastases)	+	37	35		41					
21	51	male	Renal squamous cell carcinoma	-					-	-	-	-	-
22	52	female	Renal squamous cell carcinoma	+	17	17		31	+	+	+	-	+
23	39	male	Renal squamous cell carcinoma	-					-	-	-	-	-
24	77	male	Renal squamous cell carcinoma	-					-	-	-	-	-
25	64	male	Bladder squamous cell carcinoma	+	82	82		78	+	+	+	-	+
26	61	male	Bladder squamous cell carcinoma	+	73	75	68	71	+	+	+	+	+
27	50	male	Bladder squamous cell carcinoma	+	85	87		79	+	+	+	-	+

Note: CSP, chromosomal centromeric probe; GLP, gene locus-specific probe; “+”, a positive FISH assay; “-”, a negative FISH, assay.

### 3.2 Genetic material changes in CNUL patients with positive urine FISH

Among the 25 patients included in the study, the rates of chromosome 3, 7 and 17 amplifications and GLP p16 gene deletion were 64.00% (16/25), 60.00% (15/25), 52.00% (13/25) and 8.00% (2/25), respectively. In adenocarcinoma, the amplification rates of chromosomes 3, 7, and 17 were 77.78% (7/9), 77.78% (7/9), and 44.44% (4/9), respectively, while the GLP p16 gene deletion rate was only 11.11% (1/9). In squamous cell carcinoma, the amplification rate of chromosomes 3, 7 and 17 was 54.55% (6/11), while the deletion rate of the GLP p16 gene was only 9.09% (1/11). Therefore, the incidence of GLP p16 gene deletion is very low in both adenocarcinoma and squamous cell carcinoma. There was no statistically significant difference in the frequency of chromosome 3, 7, 17 amplification and GLP p16 gene deletion in squamous cell carcinoma compared with adenocarcinoma (*p* > 0.05) (Table 2).

**TABLE 2:** Changes in genetic material of different tumour types.

Diagnosis	Chromosomal amplification/gene deletion				*P*
	3 #	7 #	p16	17 #	
Adenocarcinoma (n = 9)					*P* ^1^ > 0.05
Urachal adenocarcinoma (n = 7)	5/7 (71.42%)	5/7 (71.42%)	1/7 (14.29%)	2/7 (28.57%)	
prostate cancer (n = 2)	2/2 (100%)	2/2 (100%)	0	2/2 (100%)	
Total	7/9 (77.78%)	7/9 (77.78%)	1/9 (11.11%)	4/9 (44.44%)	
Squamous cell carcinoma (n = 11)					
Primary renal squamous cell carcinoma (n = 6)	1/6 (16.67%)	1/6 (16.67%)	0	1/6 (16.67%)	
Primary bladder squamous cell carcinoma (n = 3)	3/3 (100%)	3/3 (100%)	1/3 (33.33%)	3/3 (100%)	
Secondary renal squamous cell carcinoma (n = 2)	2/2 (100%)	2/2 (100%)	0	2/2 (100%)	
Total	6/11 (54.55%)	6/11 (54.55%)	1/11 (9.09%)	6/11 (54.55%)	
Metastatic tumour (n = 3)					
Renal squamous cell carcinoma (oesophageal squamous cell carcinoma metastasis, n = 1)	1/1 (100%)	1/1 (100%)	0	1/1 (100%)	
Renal squamous cell carcinoma (lung squamous cell carcinoma metastasis, n = 1)	1/1 (100%)	1/1 (100%)	0	1/1 (100%)	
Renal secondary non-Hodgkin lymphoma (haematologic lymphoma metastasis, n = 1)	1/1 (100%)	1/1 (100%)	0	1/1 (100%)	
Total	3/3 (100%)	3/3 (100%)		3/3 (100%)	
Other types (n = 6)					
Urothelial carcinoma (n = 2)	1/2 (50%)	1/2 (50%)	2/2 (100%)	2/2 (100%)	*P* ^2^ = 0.017
Small cell carcinoma of the bladder (n = 1)	1/1 (100%)	1/1 (100%)	0	1/1 (100%)	
Bladder paraganglioma (n = 1)	1/1 (100%)	0	0	1/1 (100%)	
Cystitis glandularis (n = 2)	0	0	0	0	
Total	3/6 (50%)	2/6 (33.33%)	2/6 (33.33%)	4/6 (66.67%)	

Note: *P* ^*1*^: compared with adenocarcinoma, there was no statistical significance in the number amplification of chromosomes 3, 7 and 17 and GLP p16 gene deletion in squamous cell carcinoma (*p* > 0.05); *P* ^*2*^: GLP p16 gene deletion rate was different between non-urothelial carcinoma and urothelial carcinoma (*p* = 0.017).

**FIGURE 1:**
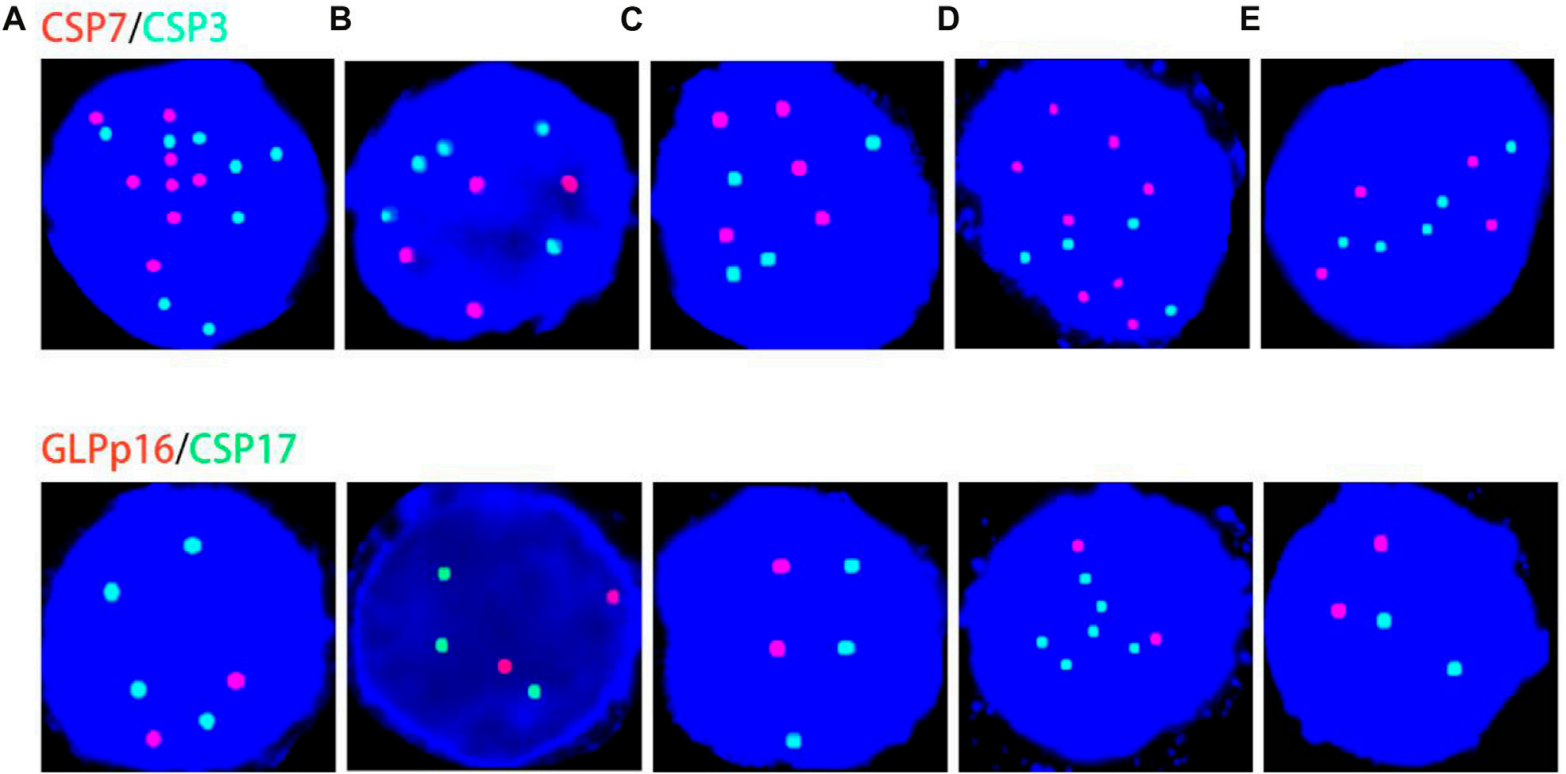
Genetic material changes in patients with distant metastases or highly malignant tumors. **(A–E)** correspond to case No. 7 (secondary non-Hodgkin lymphoma of the kidney), case No. 13 (urachal carcinoma with distant visceral metastasis), case No. 17 (renal pelvis squamous cell carcinoma derived from esophageal squamous cell carcinoma), case No. 20 (renal pelvis squamous cell carcinoma derived from lung squamous cell carcinoma metastases), and case No. 25 (primary bladder squamous cell carcinoma), respectively. Red represents CSP7 and GLP p16. Green represents CSP3 and CSP17. Note: Case B cited a case in the previous published articles of our team (Hu et al., 2020). Case A and case C cited the cases from our previous published articles of our team (Hu et al., 2021).

In addition, the proportion of cells with abnormal genetic material in urine samples of patients with bladder squamous cell carcinoma, renal secondary non-Hodgkin’s lymphoma and bladder small cell carcinoma were all greater than 65%, indicating that these tumour cells are more likely to shed into the urine (Table 1). During our data collection, we also found that for patients with advanced or distant metastases, such as renal secondary non-Hodgkin’s lymphoma, urachal carcinoma with visceral distant, metastatic renal squamous carcinoma (derived from lung squamous carcinoma and esophageal squamous carcinoma), and primary squamous carcinoma of the bladder, their urine exfoliated cells had more frequent chromosomal amplifications, often appearing as 5-ploidy and 6-ploidy (Figure 1).

### 3.3 Mutual validation of histological and urine FISH results

For this study, haematoxylin and eosin staining slides (as well as immunohistochemical slides, if applicable) of paraffin-embedded tissues of 20 patients (Paraffin sections of some patients are obsolete or have too few tissue specimens to perform histological FISH) were applied to the Pathology Department. Representative paraffin blocks containing ≥80% tumour cells were then selected for histological FISH detection, and the hybridized target area was highlighted on each representative slide.

The 20 patients included 5 adenocarcinomas (3 urachal adenocarcinomas, 2 prostate cancer invading the bladder), 10 squamous cell carcinomas (7 renal pelvis squamous cell carcinomas, 3 bladder squamous cell carcinomas), 1 bladder small cell carcinoma, 2 UC (1 bladder UC, 1 renal pelvis UC) and 2 glandular cystitis. Sections were processed in strict accordance with the instructions of the FISH kit, and *in situ* hybridization was performed using fluorescent dye-labelled GLP p16 gene locus-specific probes and CSP3/CSP7/CSP17 chromosomal centromeric probes. Pathologists with 10 years of work experience read the films.

**FIGURE 2:**
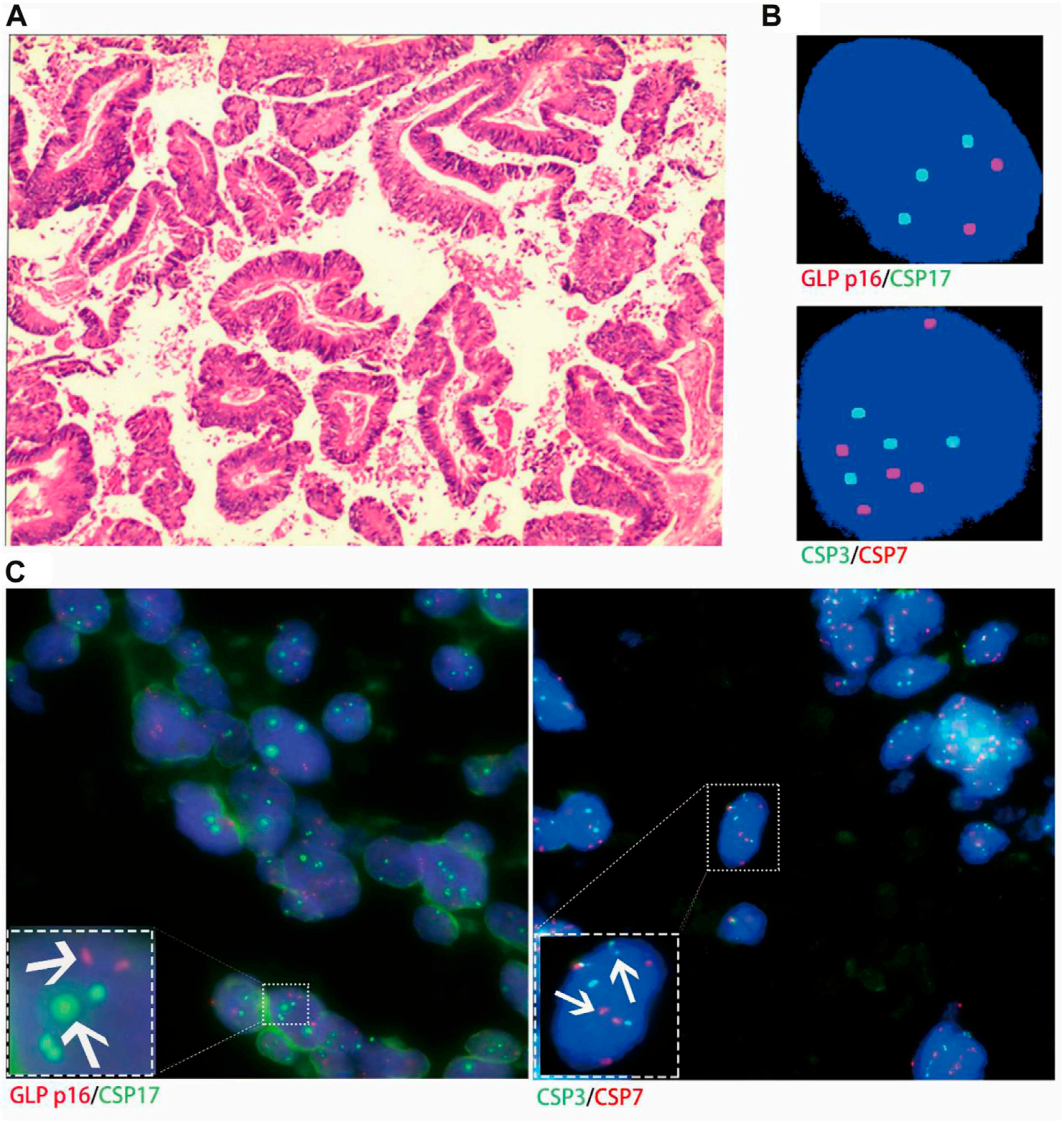
Case No.15. Positive validation of histological FISH and urine FISH in adenocarcinoma. **(A)** is the histopathological image of prostate cancer. Microscopy showed moderately and poorly differentiated with Gleason score 4 + 4 = 8(hematoxylin-eosin staining, ×200 magnification). **(B)** is the urine FISH result, showing the amplification of chromosomes 3, 7 and 17, and no GLP p16 gene deletion. **(C)** is histological FISH (×400 magnification), also showing amplification of chromosomes 3, 7 and 17, without GLP p16 gene deletion (indicated by arrows). The mean fluorescence signals of chromosome 3, 7, 17 and GLP p16 locus in each cell were 3.48, 3.94, 2.93 and 2.06, respectively. Red represents CSP7 and GLP p16. Green represents CSP3 and CSP17.

**FIGURE 3:**
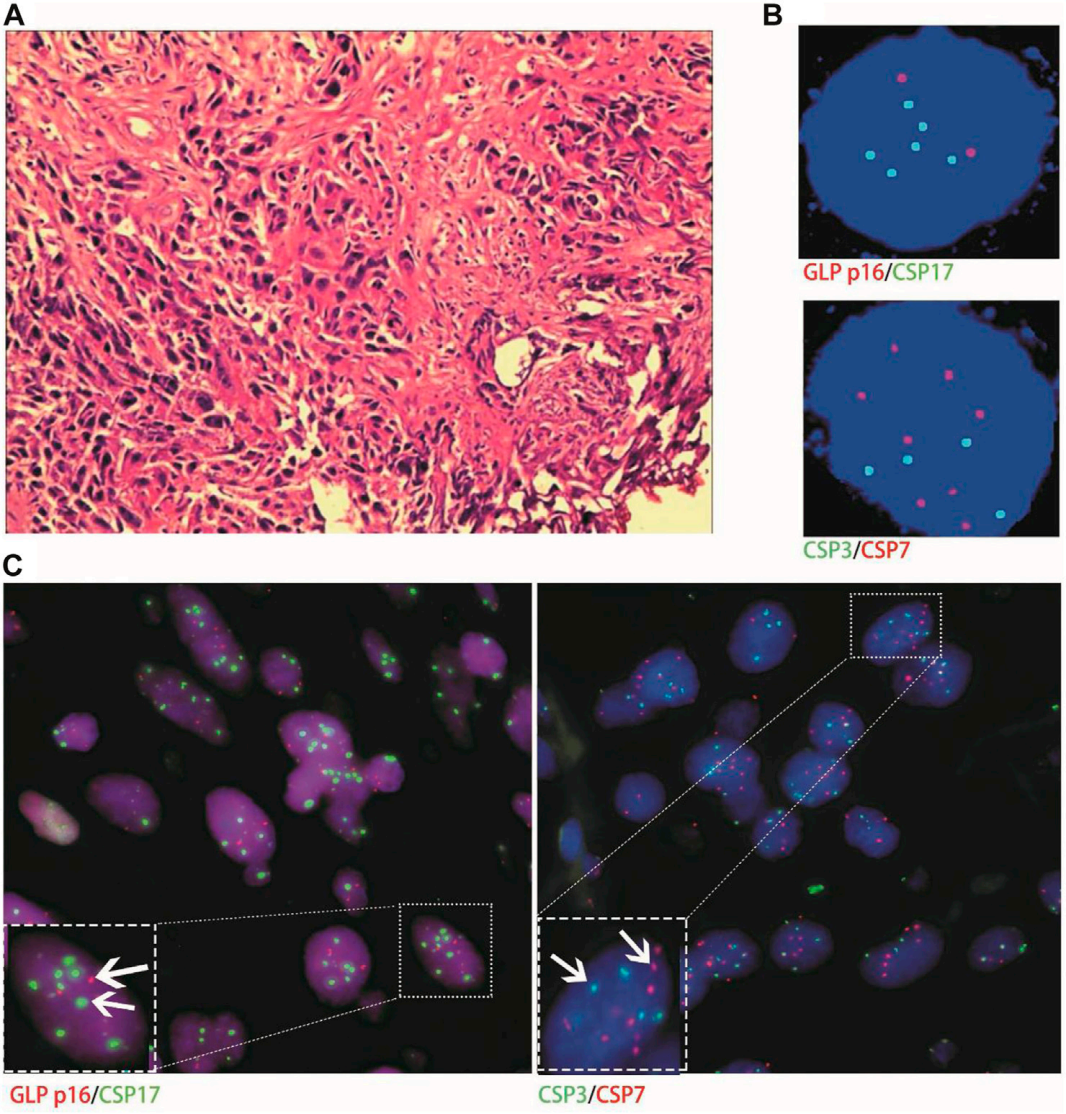
Case No.25. Positive validation of histological FISH and urine FISH in squamous cell carcinoma. **(A)** is the histopathological image of a patient with bladder squamous cell carcinoma, showing invasive squamous cell carcinoma under a microscope (hematoxylin-eosin staining, ×200 magnification); **(B)** is the urine FISH result, showing the amplification of chromosomes 3, 7 and 17, and no GLP p16 gene deletion; **(C)** is histological FISH (×400 magnification), also showing amplification of chromosomes 3, 7 and 17, without GLP p16 gene deletion (indicated by arrows). The mean fluorescence signals of chromosome 3, 7, 17 and GLP p16 locus in each cell were 3.64, 4.19, 3.86 and 1.97, respectively. Red represents CSP7 and GLP p16. Green represents CSP3 and CSP17.

**FIGURE 4:**
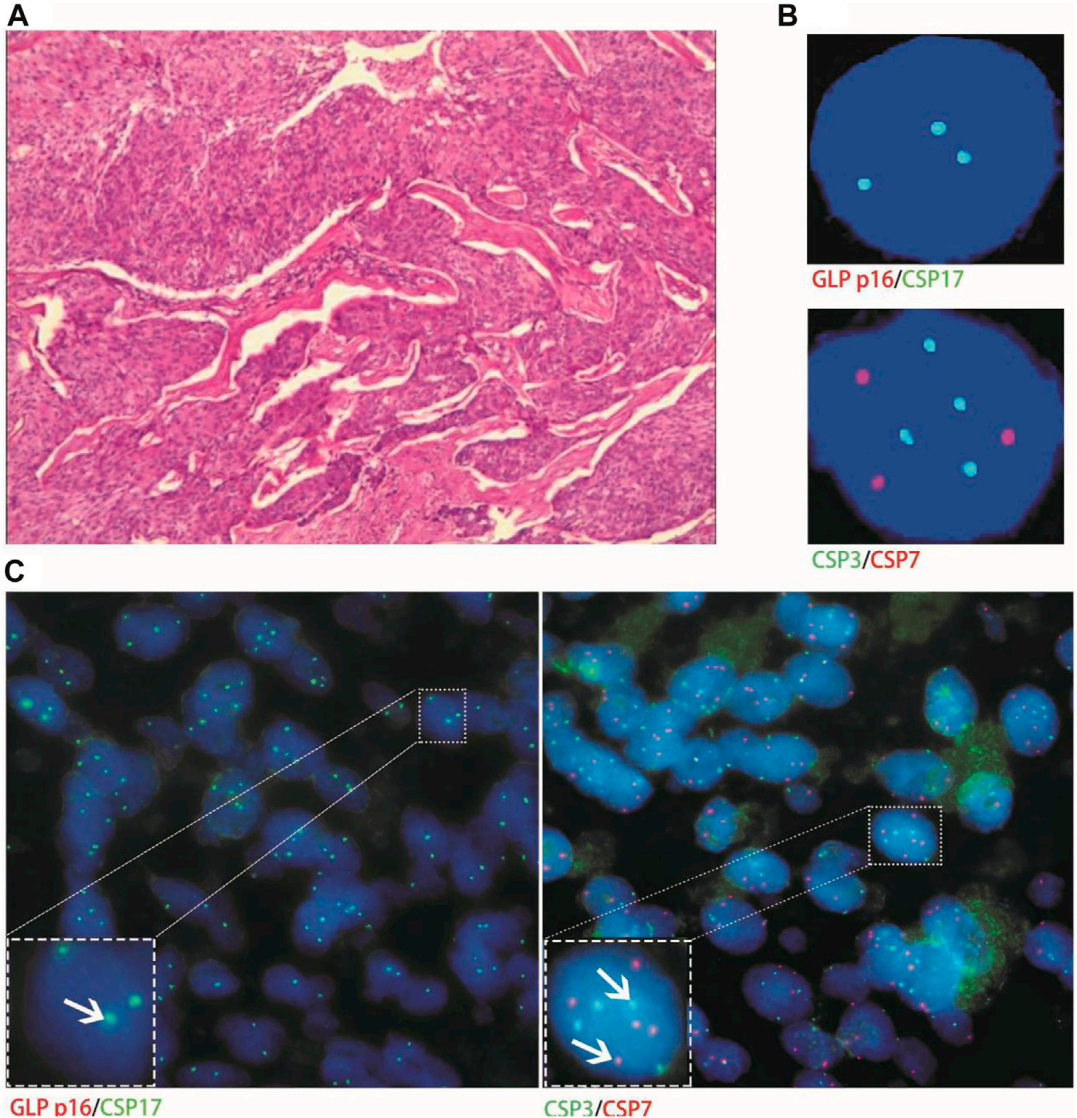
Case No.1. Positive validation of histological and urine FISH in urothelial carcinoma. **(A)** is the histopathological image of a patient with urothelial carcinoma. The microscope shows high-grade urothelial carcinoma of the bladder, invading the full thickness of the bladder wall (hematoxylin-eosin staining, ×200 magnification); **(B)** is the urine FISH, showing the amplification of chromosomes 3, 7, 17 and the deletion of the GLP p16 locus; **(C)** is histological FISH (×400 magnification), also showing amplification of chromosomes 3, 7, 17 and deletion of the GLP p16 locus (indicated by arrows). The mean fluorescence signals of chromosome 3, 7, 17 and GLP p16 locus in each cell were 3.59, 3.02, 2.87 and 0.63, respectively. Red represents CSP7 and GLP p16. Green represents CSP3 and CSP17.

**FIGURE 5:**
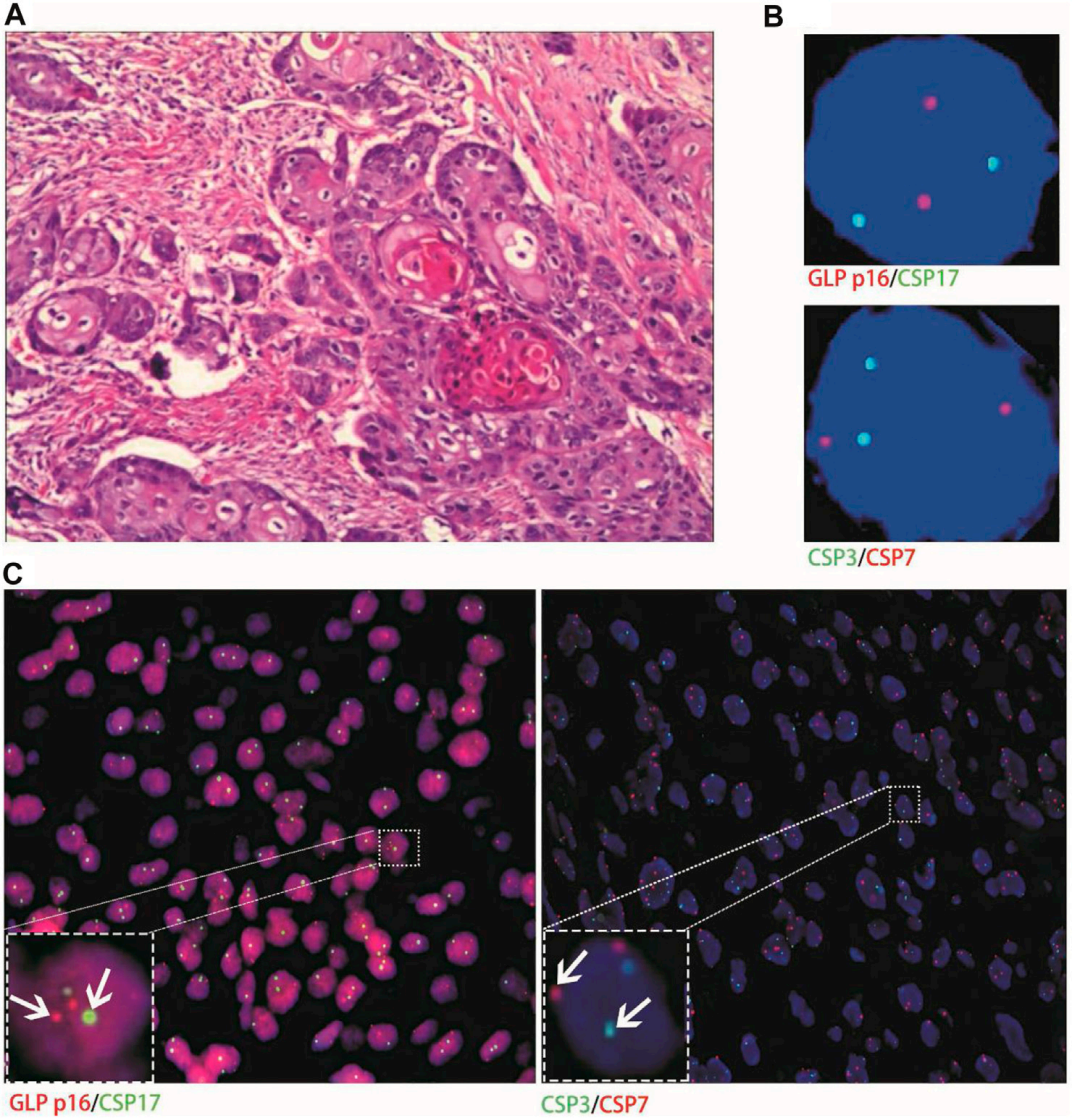
Case No.18. Negative validation of histological and urine FISH. **(A)** is the histopathological image of a patient with primary pure renal pelvis squamous cell carcinoma. Microscopically, high-medium-differentiated squamous cell carcinoma invaded perirenal fat, and no atypical hyperplasia, carcinoma *in situ*, or infiltrating carcinoma components of urinary tract epithelium were observed (hematoxylin-eosin staining, ×200 magnification); **(B)** is the urine FISH result of case No. 18, which was negative; **(C)** is histological FISH (×400 magnification), which was also negative (indicated by the arrow). The mean fluorescence signals of chromosome 3, 7, 17 and GLP p16 locus in each cell were 1.93, 2.05, 1.95 and 1.96, respectively. Red represents CSP7 and GLP p16. Green represents CSP3 and CSP17.

Histological FISH results showed that the histological results were consistent with urine FISH results in 19 patients. Due to a large number of mutually verified cases, only representative cases are shown here (one case each of adenocarcinoma, squamous cell carcinoma, and UC with positive verification and one case with negative verification of squamous cell carcinoma). Positive verification: The histological FISH of the adenocarcinoma patient in case No. 15 also showed amplification of chromosomes 3, 7 and 17 without the deletion of the GLP p16 gene, which was consistent with his urine FISH (Figure 2). The histological FISH of the squamous cell carcinoma patient in case No. 25 showed amplification of chromosomes 3, 7 and 17 and no GLP p16 gene deletion, which was consistent with the results of urine FISH (Figure 3). Histological FISH of the patient with UC in case No. 1 showed amplification of chromosomes 3, 7, and 17 and deletion of GLP p16, which was consistent with the urine FISH (Figure 4). Negative verification, such as case No. 10, indicates that histology and cytology FISH is negative (Figure 5).

**FIGURE 6:**
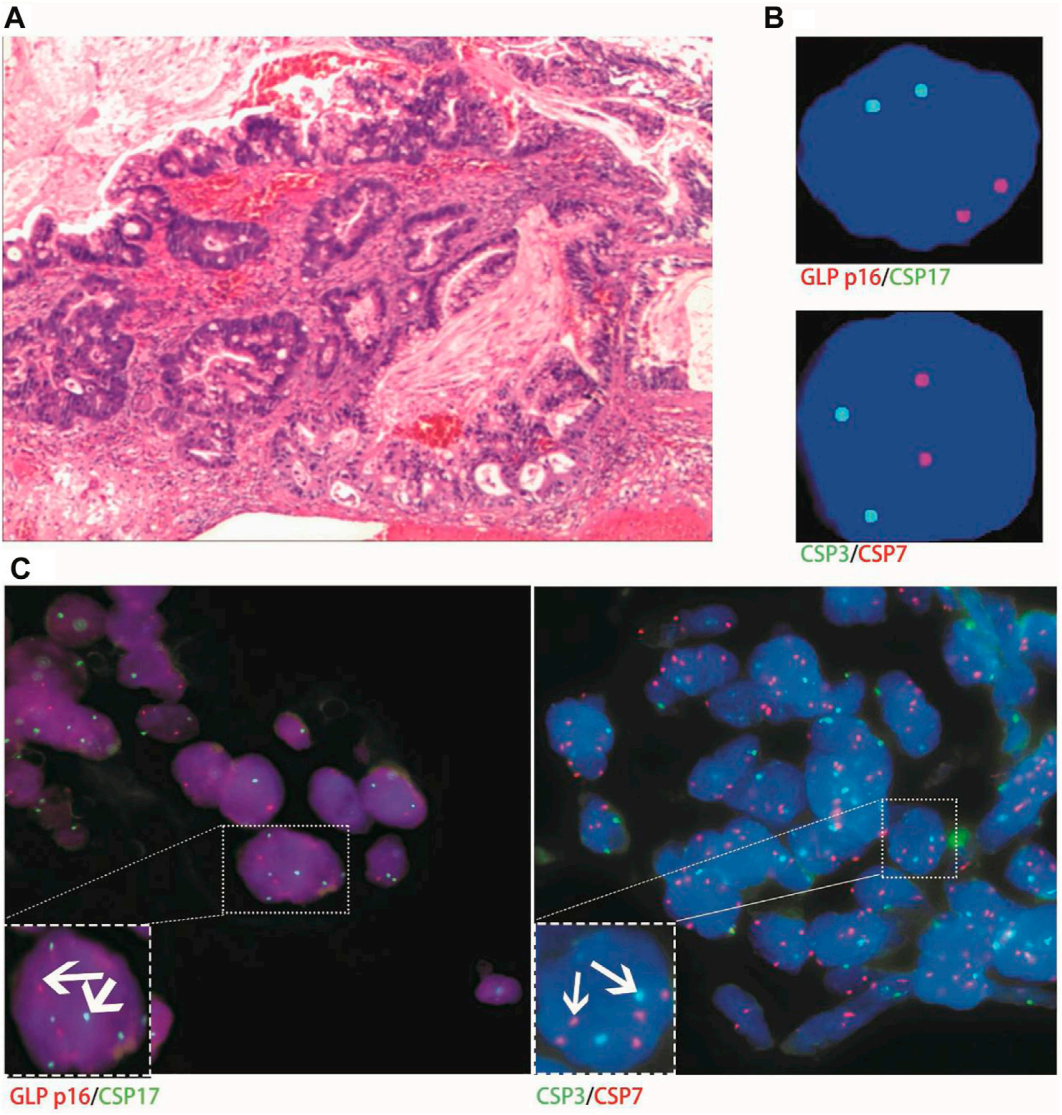
Case No.10. The histological FISH (positive) of a patient with stage Ⅲa urachal carcinoma showed amplification of chromosomes 7 and 17, which was inconsistent with its urine FISH (negative). **(A)** is the histopathological image of a patient with urachal carcinoma, showing intestinal adenocarcinoma under a microscope (hematoxylin-eosin staining, ×200 magnification); **(B)** is negative for urine FISH results; **(C)** is histological FISH (×400 magnification), showing amplification of chromosomes 7 and 17, no amplification of chromosome 3 and deletion of the GLP p16 gene (indicated by the arrow). The mean fluorescence signals of chromosome 3, 7, 17 and GLP p16 locus in each cell were 2.11, 3.88, 3.74 and 2.03, respectively. Red represents CSP7 and GLP p16. Green represents CSP3 and CSP17.

Only one patient with stage-Ⅲa urachal carcinoma had histological FISH (positive) findings of chromosome 7 and 17 amplification, which was inconsistent with its urine FISH (negative) results (Figure 6).

## 4 Discussion

Molecular cytogenetic research in recent years has focused on identifying relevant changes in abnormal cellular DNA in urine specimens, as they often precede the appearance of macroscopic and microscopic lesions, allowing detection of subclinical disease. FISH is a highly sensitive and specific molecular test for detecting urothelial carcinoma (Halling et al., 2000). However, in clinical work, the authors found that FISH also showed positive performance in urine samples of various CNUL, which aroused research interest.

Although numerous studies have evaluated the performance of FISH in typical UC, there is a significant lack of data from studies evaluating FISH in CNUL. This study found that the positive rate of FISH in adenocarcinoma was 77.78% (7/9), and there was no significant difference compared with the sensitivity of 81% in urothelial carcinoma (*p* > 0.05) (Caraway and Katz, 2010). Although the positive rate in squamous carcinoma was 54.55% (6/11), it varied widely, with 100% in both primary squamous carcinoma of the bladder (3 cases) and metastatic renal squamous carcinoma (2 cases), while the positive rate in primary simple renal pelvis squamous carcinoma was only 16.67% (1/6), so it caused us to think about it.

The low positive rate of urine FISH in pure renal pelvis squamous cell carcinoma may be due to insufficient shedding of tumor cells into the urine, or the absence of related genetic material changes such as chromosomes 3, 7, 17, and p16 genes, resulting in negative FISH. This study also confirmed that histological FISH results in patients with simple renal pelvis squamous carcinoma were consistent with urine FISH, thus suggesting that alterations in the heritage material of urine FISH-negative renal pelvic squamous cell carcinoma may not match the probe combination used. Possible reasons for the high rate of urine FISH positivity in bladder squamous cell carcinoma include the following: First, the bladder is an organ for storing urine and controlling urination. Bladder squamous cell carcinoma is a tumour of the lower urinary tract, and tumour cells are easy to shed and collect. Some studies (Gomella et al., 2017; Huang, 2020) have also shown that the sensitivity of urine FISH assay in lower urinary tract tumours is significantly higher than that of upper urinary tract tumours. Second, it is difficult for some postoperative specimens to have only one pathological type, which may or may not be accompanied by a small urothelial carcinoma component. Therefore, we will doubt whether the positive urine FISH assay is caused by UC or bladder squamous cell carcinoma. This doubt can be explained by pathological and histological FISH results. First, the pathological reports of 3 cases of bladder squamous carcinoma included in this study suggested highly differentiated squamous cell carcinoma, and no atypical hyperplasia, carcinoma *in situ* or invasive carcinoma of urinary tract epithelium was observed. In addition, the histological FISH test results of these 3 cases were consistent with urine FISH assay, suggesting that there were indeed genetic changes related to FISH positivity in bladder squamous cell carcinoma.

In a large data from multiple clinical institutions in China studied by zhou et al. (2019) suggested that UC patients with chromosome 3, 7, and 17 amplification or GLP p16 gene deletion accounted for 71.3% (2941/4125), 72.2% (2978/4125), 67.4% (2780/4125), and 72.9% (3007/4125), respectively. The changes in the genetic material in CNUL and UC patients are indeed different. CNUL patients mainly had amplification of chromosomes 3, 7 and 17 (*p* > 0.05), while the deletion rate of the GLP p16 gene was significantly lower than that of UC (8.0% vs. 72.9%, *p* < 0.001). There are many genetic abnormalities in UC during its occurrence and development. Partial or complete loss of chromosome 9 is one of the most common genetic changes. This abnormality is closely related to the early occurrence of bladder cancer because it contains important tumour suppressor genes related to cell cycle regulation (Halling et al., 2000; Sokolova et al., 2000; Zhang et al., 2004). Mutations in chromosome 9p and fibroblast growth factor receptor 3 in normal urothelium may lead to urothelial hyperplasia or low-grade papillary urothelial carcinoma, (Zhang et al., 2004) which may also explain the difference in genetic material between CNUL and UC.

During the mutual verification of histological and urine FISH, we found only one patient with stage Ⅲa urachal carcinoma whose histological FISH (positive) was inconsistent with his urine FISH assay (negative). This may be because the tumor cells were not shed in sufficient quantity in the urine, resulting in a negative urine FISH. This study can also rule out operational errors and interpretation errors and verifies that the tumour cells shed in the urine originate from tumour tissue rather than inflammatory proliferative reactions or other lesions.

The following is an analysis of why FISH is positive in urine and tissue specimens of CNUL. The FISH DNA probe used in our hospital is a combination of a centromere probe and a site-specific recognition probe provided by Beijing Jinpujia Medical Technology Co., Ltd., consisting of two combinations, CSP3 (green)/CSP7 (red) and GLP p16 (red)/CSP17 (green). If the tumor cells have chromosome 3, 7, 17 aberrations or (and) GLP p16 locus deletions and the diseased cells can be shed in sufficient quantity into the urine, both urine FISH and histological FISH are likely to be positive. Chromosomal aberrations are a prominent feature of human malignancies. Most solid tumours exhibit complex alterations of genetic material. In this study, positive urine FISH assays were found in patients with metastatic tumours and other rare and highly malignant tumours, which confirmed the findings of Ashley et al. (2006) and Lopez-Beltran et al. (Lopez-Bel et al., 2008) that patients with aggressive and highly malignant rare tumours had many genetic abnormalities. Many studies (Offit, 1992; Atkin et al., 1995; Pycha et al., 1999; Kasahara et al., 2002; Wu et al., 2006; Reid-Nicholson et al., 2009; Schaefer et al., 2010; Collazo-et al., 2016; Haisley et al., 2017; Liu et al., 2017; Reis et al., 2018) have also confirmed that adenocarcinoma (prostate cancer, urachal carcinoma), squamous cell carcinoma (bladder squamous cell carcinoma, oesophageal squamous cell carcinoma, lung squamous cell carcinoma), non-Hodgkin’s lymphoma, small cell carcinoma of the bladder, paraganglioma of the bladder, etc., may have chromosome 3, 7, 17 aberrations or (and) deletion of the GLP p16 gene locus. Therefore, FISH may be positive. For FISH-negative patients, it is possible that the genetic material changes in the tumour do not fully match the type of probe used.

This study also has limitations, such as the small number of cases collected and the lack of large multicentre samples to verify the conclusions.

## 5 Conclusion

We demonstrated for the first time the application value of FISH in CNUL on urine samples. Positive urine FISH tests indicate not only UC, but also CNUL. UroVysion^™^ FISH possibly has a high positive rate in CNUL. Urine FISH is more likely to be positive for patients with high malignancy or distant metastasis. CNUL and UC may have different genetic material changes. If a sufficient number of tumor cells are shed into the urine, the results of histological and urine FISH tests are consistent. Urologists should combine medical history and imaging information when interpreting FISH results for accurate diagnosis and treatment.

## Data Availability

The original contributions presented in the study are included in the article/Supplementary Material, further inquiries can be directed to the corresponding authors.
