# Supplementary material for: UroVysion™ fluorescence in situ hybridization (FISH) possibly has a high positive rate in carcinoma of non-urothelial lineages
Source: Front Mol Biosci. 2023 Oct 5;10:1250442. doi: 10.3389/fmolb.2023.1250442 (PMC10586327; doi:10.3389/fmolb.2023.1250442)
Supplement: Supplementary file 1 [file DataSheet1.PDF]

Ⅰ、Fluorescence In Situ Hybridization（Histology FISH testing process)[1,2]

1、Operating procedures

（1）First, the 5-μm paraffin-embedded sections were placed in a 56 ° C oven overnight and were then placed in xylene for multiple deparaffinization steps.

（2）The slides were dehydrated in 100% ethanol for 5 minutes at room temperature, and after air drying for 3 minutes, the slides were immersed in 10 mmol/L citric acid buffer (80 °C; pH 6.0) for 45 minutes, followed by immersion in 2x saline-sodium citrate (SSC) for 5 minutes at 37°C.

（3） The samples were then digested in 0.2% pepsin solution (2,500-3,500 U/mg) for 48 minutes at 37 °C, after which the sections were placed in 70%, 85% and 100% ethanol solutions. Each section was dehydrated in ethanol for 2 minutes.

（4）Next, 10 μL of the UroVysion probe (Beijing Jinpu Jia Medical Technology Co., LTD) was applied to the target tissue area. The probe and target DNA were denatured and hybridized on a HYBrite instrument at 80° C for 3 minutes and hybridized at 37° C for 16 to 18 hours. After hybridization, any unbound probe was removed by washing in 2×SSC/0.1% NP-40 (Abbott Molecular) at 76° C for 2 minutes, followed by 1 minute at room temperature in 2×SSC/0.1% NP-40. Then, 10 μL of DAPI II counterstain was applied, and the slides were cover-slipped.

2、Evaluation of results

FISH results were analysed by two professional certified double-blind pathologists with ten years of work experience. A minimum of 25 tumor cells were visualized and evaluated for these chromosomal changes. If no abnormalities were detected then the remaining cells were counted until a sufficient number of cells with chromosomal abnormalities was found or until 200 cells were evaluated. A positive result was the presence of ≥4 (or ＞10%) cells with gains of 2 or more of chromosomes 3, 7 and 17. In the case of chromosome 9 a positive result was one in which ＞12 cells showed zero 9p21 signals.

II、Fluorescence In Situ Hybridization（urine cytological specimens FISH testing process）[3]

1、Specimen processing procedures and slide preparation

（1）Fresh morning urine samples of at least 200 mL in volume were collected from the out-patients and inpatients of urology department of our hospital.

(2) Pretreatment of urine with reagents provided by Beijing Jinpujia Medical Technology Co., Ltd. The 200 mL urine sample was centrifuged at 2000 rpm for 10 min. Then, the supernatant was removed, and the pellet was re-suspended with collagenase B before incubation in a 37 °C water bath for 20 min.

(3) The sample was then centrifuged at 1000 rpm for 10 min, the supernatant was removed, and the cells were re-suspended in a 0.075mol/L KCl hypotonic solution before incubation in a 37 °C water bath for 20 min.

(4) The cells were fixed twice by the addition of 2mL fixative solution (methanol: glacial acetic acid 3:1) before centrifugation for 10 min and were transferred to a glass slide followed by aging.

(5) The slides were then treated with RNase A and pepsin, followed by rinsing in a 2× saline sodium citrate (SSC) (pH 7.0) solution and gradient dehydration in 70%, 85% and 100% ethanol that had been precooled at -20°C for 2 min.

2、FISH operation steps

（1）The slides were prepared by soaking in 73°C ± 1°C denaturation solution for 5 min, followed by gradient dehydration in 70% ethanol, 85% ethanol and 100% ethanol (-20°C) for 3 min.

(2) After natural drying, the slides were placed in a 45°C-50°C hybridization oven for 2-5 min.

(3) The probe mixtures were prepared by mixing 7 μL of hybridization buffer, 1 μL of deionized water, and 2 μL of the appropriate probe in a microcentrifuge tube at room temperature.

(4) The FISH probes were denatured at 73 °C ± 1 °C in a water bath for 5 min and stored in a 45°C-50°C water bath until the hybridization was performed. A total of 10

μL of the denatured probe mix was applied on top of the hybridization mixture on each slide. The slides were immediately covered with a cover slip and sealed with rubber cement before incubation in a wet slide box at 42°C overnight. The slides were washed immediately in 46°C± 1°C 50% formamide/2×SSC solution, 2× SSC solution, and 2×SSC/ 0.1% NP-40 solution, and then soaked in 70% ethanol in a dark box for drying. The slides were then counter-stained with 15 μL of DAPI solution. After 10-20 min in the dark room, the fluorescence signals were assessed by microscopy and images were recorded.

3. Evaluation of results

In normal cells, the CSP3, CSP7, and CSP17 centromere probes and the GLP p16 locus probe always detected a diploid state by the presence of two green and two red signals in the nucleus. The absence of a diploid signal indi cated an abnormal cell. Aneuploidies of chromosomes 3, 7, or 17 appeared as multiple signals with the nucleus whereas a missing p16 site presented as less than two fluorescent signals in the nucleus.

One hundred different cells were counted from every healthy volunteer sample, and the frequency of aneuploidies on chromosomes 3, 7, and 17 as well as the number of cells missing p16 were calculated to establish the normal threshold. When the frequency of an euploidies on chromosomes 3, 7, or 17 or the frequency of cells lacking p16 was greater than the threshold value, the sample was deemed abnormal. When the patients had at least two types of abnormal results with these probe signals, We judge the result as positive.

Ⅲ、Main reagents:

1、20×SSC, PH5.3

2、2×SSC, PH6.8~7.2

3、Denatured liquid: 70% formamide/2×SSC

4、Ethanol solution (70%, 85%, 100%) washing solution: 50% formamide/2×SSC

5、0.1% NP-40/2×SSC solution

6、Collagenase B (0.005g/5ml Hank's BSS)

7、Fixed solution (methanol: glacial acetic acid volume 3:1)

8、KCL hypotonic solution (0.075Mol KCL)

9、100μg/ml RNase A

10、20mg/ml of pepsin storage solution

11、1 Mol/L HCL

12、0.01 Mol/L HCL

13、0.1 Mol/L HCL

14、FISH DNA probe

15、CSP3/CSP7 probe

16、CSP/WPP hybridization buffer

17、GLPp16/CSP17 probe

18、GLP hybridization buffer

19、DAPI counterstain

20、Deionized water

The above reagents are mainly provided by Beijing Jinpu Jia Medical Technology Co., LTD., and the supporting reagents are provided by our hospital Reagent room supply

Ⅳ、Main equipment

1、Fluorescence microscope Japan OLYMPUS company

2、Image acquisition and analysis system Beijing Jinpujia Medical Technology Co., Ltd.

3、Heated Incubators Shanghai Xinmiao Medical Device Manufacturing Co., Ltd.

4、Low speed table top large capacity centrifuge Wuxi Ruijiang Centrifuge

5、KPJ-1A baking machine Tianjin Tianli Aviation Electromechanical Co., Ltd.

6、BCD-219D refrigerator Qingdao Haier Co., Ltd.

7、Other Centrifuge tube (15mL, 50ml) Tip Pipette sealing glue thermometer Coplin bottles, etc.

References

[1] Reid-Nicholson MD, Ramalingam P, Adeagbo B, Cheng N, Peiper SC, Terris MK. The use of Urovysion fluorescence in situ hybridization in the diagnosis and surveillance of non-urothelial carcinoma of the bladder. Mod Pathol. 2009;22(1):119-27

[2] Kipp BR, Tyner HL, Campion MB, Voss JS, Karnes RJ, Sebo TJ, et al. Chromosomal alterations detected by fluorescence in situ hybridization in urothelial carcinoma and rarer histologic variants of bladder cancer. Am J Clin Pathol. 2008;130(4):552-9.

[3] Zhou L, Yang K, Li X, Ding Y, Mu D, Li H, et al. Application of fluorescence in situ hybridization in the detection of bladder transitional-cell carcinoma: A multi-center clinical study based on Chinese population. Asian J Urol. 2019;6(1):114-21.
